# Surgical management of ventricular septal rupture post device migration: a case report

**DOI:** 10.1186/s13019-026-03864-w

**Published:** 2026-02-03

**Authors:** Vivek Jaswal, Sanketh Edem, Anand Kumar Mishra, Akshay Kheni, Lasya Reddy

**Affiliations:** Department of Cardiothoracic and Vascular surgery, Post Graduate Institute of Medical Education and Research, Chandigarh, India

**Keywords:** Posterior ventricular septal rupture, Inferior wall myocardial infarction, Percutaneous closure, Surgical repair, Device migration, Tricuspid valve injury, Infarct exclusion, Residual shunt

## Abstract

**Background:**

Ventricular septal rupture (VSR) following myocardial infarction is a rare but life-threatening complication. While percutaneous closure offers a less invasive option, it remains technically challenging in posterior defects due to anatomical constraints. This case report emphasizes the limitations of device-based closure in such settings and reinforces the role of surgery as the definitive management.

**Case presentation:**

: We report the case of a 58-year-old male who developed a posterior ventricular septal rupture following an acute inferior wall myocardial infarction. Initial management included percutaneous device closure of the VSR. However, the patient developed persistent residual shunting and device migration, which resulted in its entanglement with the tricuspid valve apparatus and worsening heart failure. The patient subsequently underwent successful surgical repair through device extraction, infarct exclusion technique along with tricuspid valve replacement. Postoperative recovery was uneventful, and follow-up echocardiography confirmed complete closure of the defect with preserved biventricular function.

**Conclusions:**

This case underscores the limitations of percutaneous closure in posterior VSRs and supports surgical repair as the more reliable and definitive treatment. This case is unique due to extensive device migration into the tricuspid valve apparatus, necessitating combined device extraction, infarct exclusion repair, and tricuspid valve replacement—an uncommon combination in posterior VSR. Medium-term follow-up demonstrated stable cardiac function and no residual shunting.

## Background

Post-myocardial infarction ventricular septal rupture (VSR) is a rare but life-threatening complication, with an incidence of 0.17–0.31% following MI [1]. Advances in interventional cardiology have led to the development of percutaneous closure devices as an alternative to surgical repair. However, device closure has limitations, including malposition, residual shunting, and interference with adjacent structures, often necessitating surgical revision [2]. Major contemporary guidelines recommend early surgical intervention as the definitive management for post-MI VSR [3, 4]. This report details a case of failed device closure requiring surgical intervention. Posterior VSRs are particularly challenging for percutaneous closure because of deficient inferior rims, their close proximity to the tricuspid valve apparatus, and the dynamic geometry of the inferobasal septum. These anatomical features significantly increase the risks of device malposition, migration, and residual shunting. Although percutaneous closure remains a useful strategy in carefully selected anterior defects or as a bridge in critically ill patients, posterior defects usually require definitive surgical repair. This case is noteworthy due to the unusual extent of device entanglement in the tricuspid valve, ultimately necessitating tricuspid valve replacement in addition to infarct exclusion repair.

## Case presentation

A 58-year-old male, chronic smoker, and alcoholic with no known comorbidities or family history of coronary artery disease presented to the medical emergency department with sudden onset chest pain and palpitations. He was admitted to the cardiology ICU with a pulse rate of 104/min, blood pressure was 90/60 mmHg, and oxygen saturation was 92% on room air. Chest auscultation revealed a holosystolic murmur at the left lower parasternal border, and bilateral lung crepitations were noted.

Electrocardiography showed ST elevation in leads II, III, and aVF, suggestive of an inferior wall MI. Transthoracic echocardiography revealed inferior wall hypokinesia, an ejection fraction of 40–50%, and a 27 mm mid-muscular ventricular septal rupture with a left-to-right shunt (gradient of 45mmHg). Coronary angiography revealed complete occlusion of the mid-right coronary artery (RCA), a plaque in the proximal RCA, and an 80% stenosis in the mid-left anterior descending (LAD) artery. The patient underwent percutaneous coronary intervention (PCI) with stenting of the LAD using an Ultimaster Nagomi stent, restoring TIMI III flow.

**Fig. 1:**
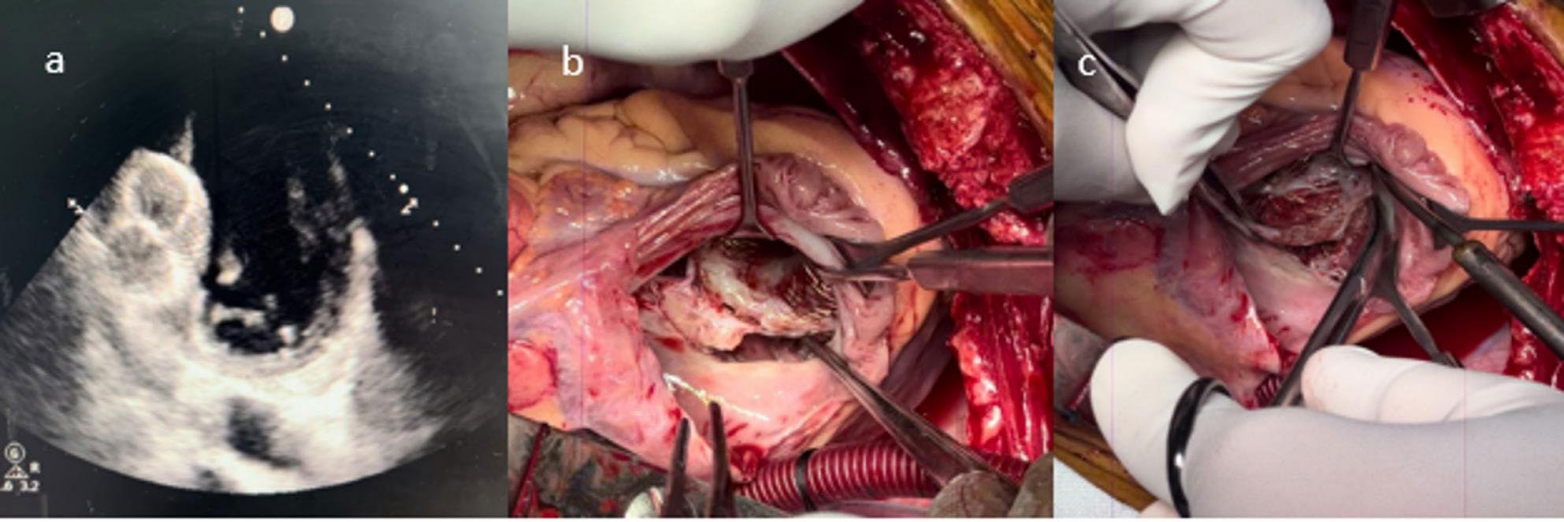
**a**) 2d Echo showing migrated device, **b, c** device densely adhere to the tricuspid valve

Following the intervention, the patient developed acute heart failure with a Pro-BNP level of 3536 ng/dL. Despite medical management, the patient was still in heart failure, prompting percutaneous closure with a Life-Tech 40 mm atrial septal defect (ASD) device seven days post-MI. Post-procedural echocardiography showed an 8 mm residual VSR with a left-to-right shunt, and the patient continued to experience recurrent heart failure episodes [5]. (Fig. 1a) Although the surgical team recognized that posterior VSRs generally have poor outcomes with device closure, the interventional cardiology team strongly favored attempting percutaneous therapy, reflecting both enthusiasm for evolving transcatheter techniques and the desire to avoid high-risk early surgery [2, 6]. Echocardiography at that time was interpreted as showing a mid-muscular defect with potentially acceptable rims, and the heart team ultimately proceeded with device closure as a temporizing measure. From the surgical standpoint, this approach was undertaken with caution, acknowledging the anatomical limitations and the likelihood that definitive surgical repair would still be required.

CT thorax revealed malposition of the device, which appeared dumbbell-shaped with most of its structure in the right ventricular (RV) lumen. The superior portion of the device was located in the left ventricle (LV), abutting the interventricular septum. The RV part of the device had also become densely intertwined with the septal tricuspid leaflet (STL) and its chordae, causing rupture of its chordae and adherence to the anterior wall of the RV and the papillary muscle of the STL [7]. Due to these complications, surgical intervention was deemed necessary.

The patient underwent surgery 12 weeks after the initial MI. A median sternotomy was performed, and cardiopulmonary bypass (CPB) was initiated via aortobicaval cannulation. Right superior pulmonary vein (RSPV) and pulmonary artery (PA) vents were placed, and antegrade Del Nido aortic root cardioplegia was administered after cross clamping the aorta. The right atrium (RA) was opened, and dumbbell shaped device was found embedded in the RV with one end extending into the LV. The RV end of the device was firmly entangled with the STL and its chordae, leading to significant structural damage of the tricuspid valve [8] (Figure 1b and c).

**Fig. 2:**
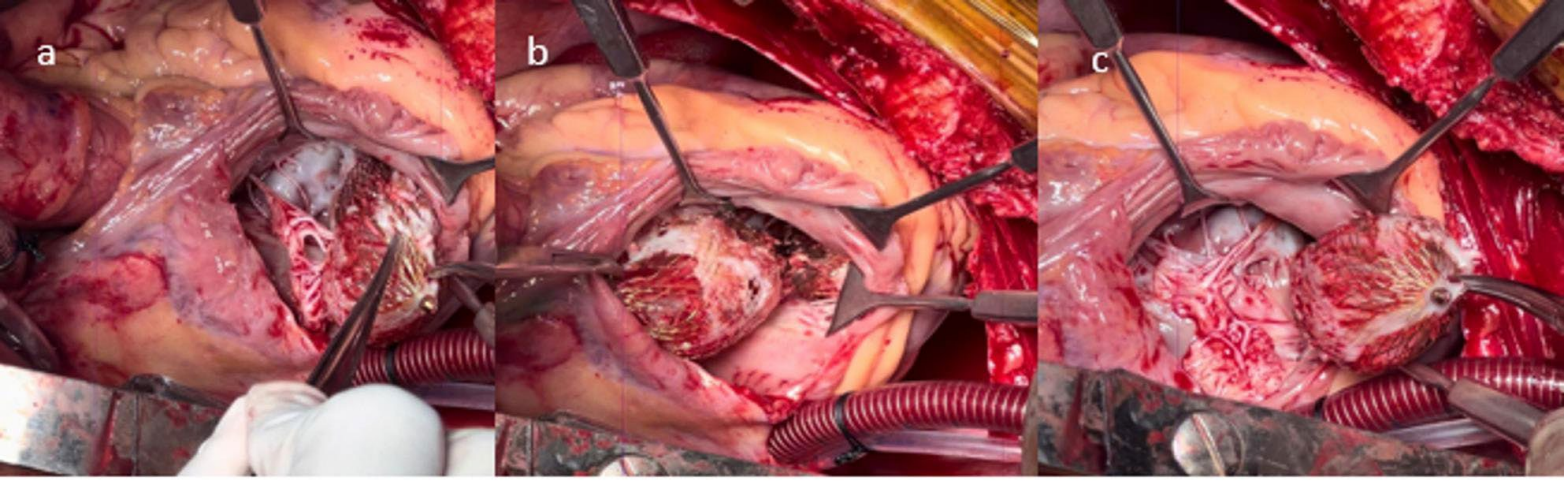
**a, b, c) **Device densely adhere to the papillary muscles of tricuspid valve

**Fig. 3:**
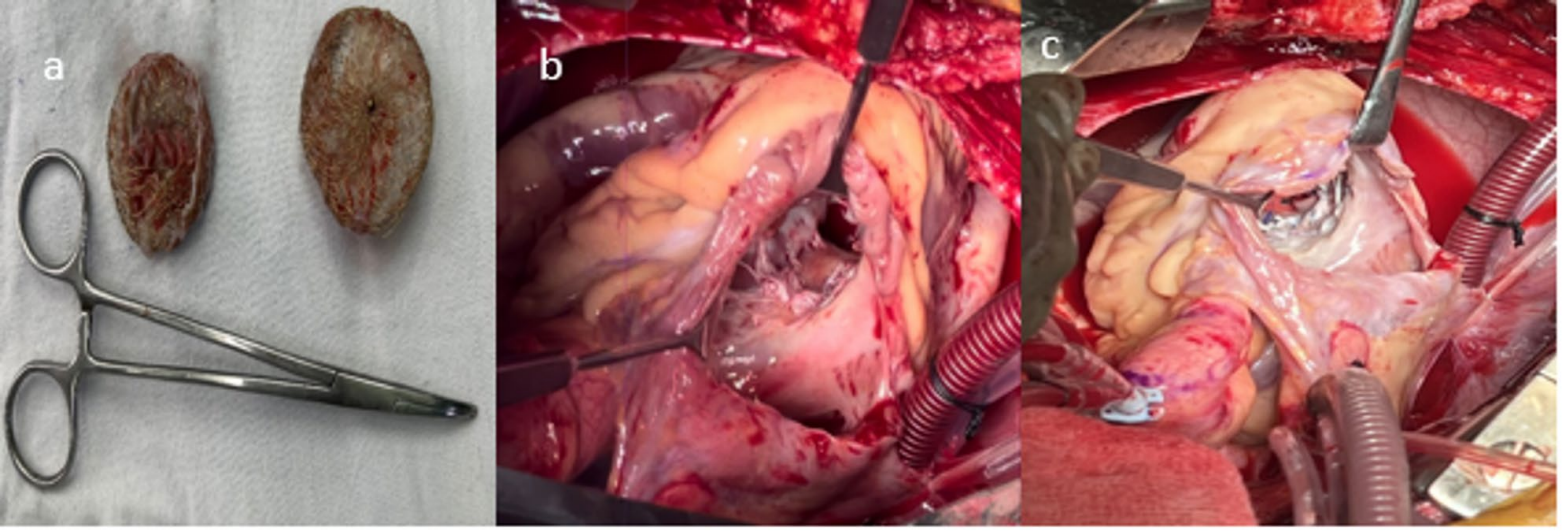
**a**) Extracted device **b**) 27x25mm mid muscular venticular septal defect **c**) defect closed with Dacron patch with 4-0 prolene in 2 layers

Careful dissection was performed to excise the STL and device from the surrounding attachments. Only limited excision of friable septal muscle was performed, strictly for the purpose of releasing the embedded device and its adherent margins (Fig. 2). This minimal debridement did not enlarge the defect and remained consistent with the principles of infarct exclusion repair. The device was extracted from the LV by excising its marginal attachments along the walls of the VSR (Fig. 3a). The VSR was then closed using a single Dacron patch on the RV side with 4 − 0 prolene sutures in 2 layers in continuous manner (Figure 3b and c). The patch was sutured to healthy muscle around the VSR. Due to the extensive tricuspid valve damage, the septal tricuspid leaflet (STL) was excised, and the tricuspid valve was replaced with a 29 mm Meril FloMech bioprosthetic valve using 13 pledgeted 2 − 0 Ethibond sutures. The RA was closed in two layers (Figure 2a, b and c).

Postoperative Transesophageal echocardiography (TEE) post-CPB revealed a left ventricular ejection fraction (LVEF) of 30–35% and a right ventricular ejection fraction (RVEF) of 30–35%. The VSR closure was successful, with no residual shunt, no mitral regurgitation, and a tricuspid valve mean gradient of 1mmHg without paravalvular leak [9]. The patient was extubated on post-operative day 2, shifted out of surgical ICU on post-operative day 5 and discharged in stable condition on post-operative day 8. The patient has been on regular follow up for 11 months now Echocardiography demonstrated a well-functioning bioprosthetic tricuspid valve with a mean gradient of 1 mmHg, preserved biventricular function and no residual septal defect.

## Discussion

Ventricular septal rupture (VSR) following acute myocardial infarction (MI) continues to pose a formidable clinical challenge, with high morbidity and mortality despite advances in interventional and surgical techniques. While percutaneous device closure has emerged as a less invasive alternative to surgical repair, its limitations—particularly in posterior VSRs—highlight the continued superiority of surgical intervention in many cases.

Percutaneous closure, though attractive for its minimally invasive nature, carries significant risks in posterior VSRs due to anatomical constraints [2, 10–12]. Percutaneous closure has been used in three principal scenarios: (1) as an acute stabilizing measure within the first 3–5 days post-MI in patients unsuitable for surgery, (2) in the subacute phase after partial tissue remodeling, when septal rims become more supportive for device anchoring, and (3) as a salvage therapy for residual or recurrent shunts following surgical repair [2, 5, 10, 12]. Appropriate patient selection requires careful assessment of rim quality, defect size, ventricular geometry, and proximity to valvular structures. Importantly, device closure of patch leaks after surgical repair yields far higher procedural success and considerably lower 30-day mortality compared with primary device closure of de novo VSR [2, 10, 13]. This distinction is clinically relevant and should guide decision-making. Inferior wall MI-associated VSRs often exhibit deficient inferior rims, making device anchoring unstable and increasing the likelihood of malposition, embolization, or residual shunting [10]. The current case, where a migrated device became entangled in the tricuspid valve apparatus, underscores this risk and, in our case, resulted in destruction of the tricuspid valve leaflet requiring valve replacement, which, to our knowledge, has not been reported previously. Studies confirm that posterior VSRs have higher procedural failure rates and higher mortality with percutaneous closure compared to anterior defects, frequently necessitating conversion to surgery [5, 6, 8, 10]. Additionally, posterior defects often require larger occluders, which may further destabilize the septum and increase the risk of mechanical complications, including valve interference and ventricular remodeling [12]. Comprehensive pre-procedural imaging is essential for selecting appropriate candidates for device closure. TTE and TEE define defect morphology, rim adequacy, and associated valvular involvement. Cardiac CT with 3D reconstruction offers enhanced spatial characterization, particularly in posterior VSRs, and can aid in device sizing. Emerging modalities such as 3D echocardiography and patient-specific 3D printing may further improve procedural planning for anatomically complex cases.

Surgical repair offers definitive treatment for posterior VSR by allowing direct visualization and secure closure of the defect. The ability to perform infarct exclusion techniques (e.g., patch repair with resection of necrotic tissue) not only ensures a durable closure but also mitigates the risk of progressive ventricular dysfunction. Moreover, concomitant procedures—such as tricuspid valve repair or CABG—can be performed in the same setting, addressing secondary complications that percutaneous approaches cannot [14]. 

In the current case we, have closed the VSR using a single patch on the RV side in contrast to classical 2 patch technique. It is much akin to the congenital VSD closure except the sutures were taken slightly farther on the surrounding healthy RV muscle. Our approach was from the right atrium, avoiding a ventriculotomy and its complications. This saves us crucial aortic cross-clamp time and CPB time in view of the patient’s age and pre-operative condition, and contributed to an excellent post-operative outcome.

Clinical evidence supports lower long-term mortality with surgical repair in posterior VSR, particularly in hemodynamically unstable patients. Device closure, while advantageous in select anterior VSRs, is associated with higher residual shunt rates (up to 30–40% in some series), which can precipitate heart failure and necessitate reintervention. Surgical closure, by contrast, provides more complete and durable sealing, reducing the need for repeat procedures.

Observational series suggest that larger defects (typically > 15 mm) are associated with lower device stability, more residual shunting, and worse survival, making surgical repair the preferred strategy in most such cases (Table 1). 

Percutaneous closure may be appropriate for small, well-circumscribed defects—most commonly anterior VSRs—with adequate rims, particularly in high surgical-risk patients or when used as a bridge to surgery. In contrast, large (> 15 mm) posterior or inferobasal defects, especially those adjacent to valvular structures, have consistently demonstrated poorer device stability and higher complication rates. Therefore, surgical repair remains the preferred definitive treatment for posterior VSRs, as exemplified by the present case.

**Table 1:** Summary of key studies reporting outcomes of percutaneous device closure for Post-Myocardial infarction ventricular septal rupture

Study (First Author)	Journal/Year	*n* (Patients)	Main Population/Design	Key Findings
Calvert et al.	Circulation, 2014	53	Multicentre UK experience of transcatheter closure for post-MI VSR	Device implant success 89%; 58% survived to hospital discharge; residual shunt common.
Assenza et al.	Circ Cardiovasc Interv, 2013	30	Single-centre experience using double-umbrella devices	Feasible closure; mortality remained high; MELD-XI score predicted death.
Premchand et al.	Indian Heart J, 2017	7	Single-centre Indian experience using ASD-type occluders	Device success 71% (5/7); 29% long-term survival; poor outcomes in inferior MI with shock.
Wilson & Horlick	EuroIntervention, 2016	Review	Narrative review of post-MI VSR management	Summarizes selective role of transcatheter closure; emphasizes surgery for posterior defects.

Data extracted from Calvert et al., Premchand et al., Assenza et al., and Wilson & Horlick

## Conclusion

While device closure offers a less invasive option in selected anterior VSRs or as a stabilizing measure in critically ill patients, surgical repair remains the treatment of choice for posterior VSRs due to its superior durability, lower complication rates, and ability to address associated cardiac pathology and valvular injury. The present case underscores the limitations of device therapy in posterior defects and highlights the durability of surgery as the preferred strategy.

## Data Availability

Data available on request from the Authors.
